# Annotations

**Published:** 1905-05-20

**Authors:** 


### ANNOTATIONS.

### Difficulties under the Infectious Diseases Notification Act.

The practitioner anxious faithfully to discharge the responsibilities inflicted on him by law in reference to the notification of cases of infectious disease has in occasional instances no light task on his hands. The difficulties of confident and early diagnosis are well-known, and on several occasions appeal for assistance to the medical officer of health has met with anything but a sympathetic response. Whether it is or is not the duty of this official to afford help and share responsibility in cases of doubtful diagnosis, there need be no hesitation in saying that the issue of a formal certificate of notification arranged as it is for the protection of the public ought to be taken by some public officer as the responsible agent of the community. The present arrangement compels the private medical adviser, who is, and ought to be, concerned with the welfare of the individual patient suddenly to act on behalf of the public, but whilst so acting, he, and not the community which he represents, is left to bear the whole responsibility. If the practitioner elects, as in the interests of his patient he may be fully justified in doing, to wait until the diagnosis is entirely beyond dispute there may be risk to the health of the neighbourhood and the practitioner may find himself liable to proceedings in a police court. On the contrary, if he decides to act on a suspicion more or less strong—and this may be highly advisable in the public interest—he runs the risks which attach to a mistaken diagnosis and an unwarranted removal of the patient. It ought to be sufficient for the practitioner to intimate to the authorities that he has reason to suspect the existence of some form of infectious disease, and all subsequent steps conceived in the public interest should be taken as official action and on official responsibility. Were such a policy adopted, not only would it be more fair to the private practitioner but the early diagnosis and prompt isolation which are so necessary to prevent epidemic extension would be secured; and such undignified squabbles as the one which has recently appeared in the press would be altogether avoided.

### Science Degrees for Pharmacists.

The Scottish Universities, following in this respect the lead of some of their sister corporations in the south, appear to be lending a favourable ear to the request of the pharmacists for the establishment of science degrees in pharmacy. Thus the Senatus of the University of Glasgow has requested the University Court to frame an ordinance for carrying out a scheme for a university curriculum for pharmaceutical students and for the institution of the degree of B.Sc. in pharmacy. To this request the Court has expressed its willingness to consent, but has at the same time hinted the difficulty which attends so many proposals—namely, the absence of funds. A suggestion that the Pharmaceutical Society might do something to endow a lectureship or demonstratorship in] pharmaceutics has not advanced matters, as the Society in turn has replied that it has no funds available for such a purpose, and, further, that pharmacists ought not to be expected to endow lectureships any more than medical practitioners are expected to contribute to the establishment of professorial chairs in the several departments of the medical curriculum. The difficulty would be solved if that remedy of the impecunious educational reformer north of the Tweed, namely, the Carnegie Trust, could be applied, but whether this can be made available remains to be seen. In the meantime, a scheme of extramural teaching recognised by the University is proposed. In any event, it would be an advantage to medical as well as to pharmaceutical students were a thoroughly competent university teacher of pharmacy appointed. Professors of Materia Medica do not always satisfy this condition, and the so-called practical classes in the subject are, in not a few instances, of little or no value. The responsibility for the decay of efficient prescribing, of which so much is heard in modern days, rests very largely with the inefficiency of the medical schools in this department.

### Common Sense in Education.

At the annual meeting of the Childhood Society on the 10th inst. at the house of the President, Earl Egerton of Tatton, an address was given by Professor H. E. Armstrong on the teaching of the rare faculty known as "common sense" in the school of the future. Professor Armstrong thinks that common sense is sadly needed in the school of to-day, where the education is altogether too bookish, and where the pupils are not sufficiently invited to exercise their own intelligence, but are compelled to get up, more or less unwillingly, subjects in which they can see no use. Unless a change in method is adopted in all our schools, from the universities downwards, he anticipates that Oxford and Cambridge will survive as ancient monuments only. That efforts are being made to repair the deficiencies of our present system he admits, but holds that they are far too feeble to be of much avail. For one school only has the professor unqualified praise. That is Osborne College, where, under Sir John Fisher's guidance, one-third of the time is devoted to literary studies, one-third to mathematics, and one-third to the workshops. If it be objected that this is the education of sailors, the sooner we are all educated like sailors the better. It is his readiness and versatility that make the sailor the "handy man," and what is necessary in our schools is that workshop methods should be substituted for didactic desk methods. How ineffective these are is proved by the fact that among hundreds of pupils, hardly one knows how to read, in the sense of being able to get up a subject from books. To remedy this it would be necessary to put practical persons in charge of our schools, and to teach children not merely to learn but to do. Practical work is enormously valuable as a moral and intellectual training. The common-sense training of the future would be founded on Carlyle's saying. "Man is a tool-using animal." If the children are taught to exert themselves, common sense will come as a matter of course.

